# Phytoestrogens and Breast Cancer: Should French Recommendations Evolve?

**DOI:** 10.3390/cancers14246163

**Published:** 2022-12-14

**Authors:** Aurore Mauny, Sébastien Faure, Séverine Derbré

**Affiliations:** 1 Department Pharmacy, Faculty of Health Sciences, University of Angers, F-49000 Angers, France; 2 Inserm, CNRS, MINT, SFR ICAT, University of Angers, F-49000 Angers, France; 3 SONAS, SFR QUASAV, University of Angers, F-49000 Angers, France

**Keywords:** soy, breast cancer, isoflavones, phytoestrogens, mortality, recurrence

## Abstract

**Simple Summary:**

Health agencies in several countries have different recommendations regarding the consumption of soy isoflavones (phytoestrogens) in breast cancer (BC) survivors. This review is an analysis of prospective cohort studies published between 2009 and 2020 to determine whether there is a rationale for advising avoidance of soy in dietary supplements and/or foods in women with a history of BC. The endpoint was breast cancer recurrence and/or mortality, and the association with soy isoflavones intake was specifically targeted. No negative effect of soy isoflavones on BC mortality/recurrence was found. These natural products could have beneficial effects. These results coincide with other recent work and suggest that taking soy isoflavones is safe for breast cancer survivors. These data no longer seem to coincide with the French recommendations, which may need to be changed.

**Abstract:**

Breast cancer (BC) occurs less frequently in Asia, where there is high soy consumption. It has been hypothesized that soy isoflavones could be protective against BC recurrence and mortality. At the same time, health organizations in several countries have differing recommendations for soy consumption (soy foods or dietary supplements) in BC survivors. The objective of this review is to analyze the literature and to determine whether it is justified to advise avoiding soy in dietary supplements and/or food in women with a history of BC. We conducted a systematic literature search with the Medline/Pubmed and Web of Science databases. Only prospective cohort studies published since 2009 were retained. The endpoint of studies was BC recurrence and/or mortality, and the association with soy isoflavone intake was specifically targeted. Seven studies were included. None of these studies found statistically significant adverse effects of soy consumption on BC recurrence or mortality (specific or all-cause). Overall, only one study was not able to find beneficial effects of soy intake on BC patients. The other studies concluded that there were positive associations but in very variable ways. Two studies found a decrease in BC recurrence associated with a higher isoflavone intake only for post-menopausal women. The other four studies concluded that there were positive associations regardless of menopausal status. Four studies showed better results on women with hormonal-sensitive cancer and/or patients receiving hormonal treatment. Only one found a stronger association for patients with ER-negative BC. No adverse effects of soy isoflavones on BC mortality/recurrence were found. Soy isoflavones may exert beneficial effects. These results coincide with other recent works and suggest that soy isoflavone intake is safe for BC survivors. Thus, these data no longer seem to coincide with the French recommendations, which could then be brought to evolve. However, in order to confirm the current results, larger studies are needed.

## 1. Introduction

Breast cancer (BC) is the second most common cancer in the world and the most frequently diagnosed in females. There are about 2.3 million new cases and more than 680,000 deaths each year [1].

After the initial treatment, women may experience a recurrence of the disease. Many factors may influence the risk of relapse. The characteristics of the cancer are the main factors that influence the risk of recurrence. Tumors of high grade, large size, and axillary nodal involvement have been shown to increase the risk of locoregional recurrence or metastasis. Women with a pre-menopausal diagnosis of BC have a greater risk of recurrence [2]. The tumor characteristics (high grade, high tumor size, and nodal involvement) are also associated with a higher risk of death and BC recurrence. Conversely, those risks are lower for patients with estrogen- or progesterone-receptor-positive tumors than in estrogen- or progesterone-receptor-negative ones. Women affected by this type of cancer are treated by hormonotherapy most of the time. This treatment is used to reduce the risk of relapse. Lifestyle factors might also influence the risk of recurrence and death after BC diagnosis. Excess weight is associated with both an increased risk of cancer and a higher risk of recurrence and death. Similar findings have been reported for both alcohol and tobacco use [3].

The BC incidence rate is significantly higher in Western countries than in East and Southeast Asia [1]. One assumption put forward to explain this is the difference in lifestyle and diet, such as the high consumption of soy products in East Asia, associated with increased intake of phytoestrogens (Japan and China) [4].

Due to their structural similarity with 17β-estradiol, phytoestrogens can bind to estrogen receptors (ER) and exert estrogenic effects. Indeed, they are selective ER modulators (SERM). That means they have a preferential selectivity for ERβ compared to Erα, while the mammary gland mainly contains Erα [5]. The repartition of these subtypes varies according to the types of tissues, which could influence the cellular response to different phytoestrogens (Table 1). Thus, ERα activation in mammary tissue enhances cell proliferation, which may contribute to the development of ER-dependent breast tumors. ERβ activation plays a role in counteracting ERα-mediated cell proliferation [6,7].

Moreover, phytoestrogens also exert non-estrogenic effects. In vitro, genistein has epigenetic effects that may influence the regulation of cell proliferation and differentiation. This mechanism may affect BC development. Genistein has an inhibitive effect of protein kinase that might prevent the development of breast tumor cells [4].

Phytoestrogens are nonsteroidal naturally occurring plant compounds. Five major classes of phytoestrogens have been described according to their chemical structures. Most of them are polyphenols, among which, besides isoflavonoids, some lignans, coumestans, (prenylated) flavonoids, and stilbenes were described as phytoestrogens. Some of them are found in cultivated edible or medicinal plants [6,8,9].

Soybean (*Glycine max* (L.) Merr.) is a food plant rich in isoflavones. Isoflavones are also found in beans, chickpeas, hops, red clover, kudzu, and lentils. The main molecules of this group are genistein (**1**), daidzein (**2**), and glycetin (**8**), which account for 50%, 40%, and 10%, respectively, of the total soybean isoflavone content [7]. Soy-derived products are the only common foods that contain relevant amounts of isoflavones. Naturally, in plants, most isoflavones are glycoside derivatives (e.g., genistein 7-glucoside (**1′**)). This means the isoflavone part, called aglycone, is bound to a sugar via a glycosidic bond. Isoflavone glycosides do not exert any estrogenic effect, while isoflavone aglycones are biologically active. The proportion of these two forms, i.e., isoflavone aglycones or glycosides, varies among different kind of foods. For soy foods, it mainly depends on the processing conditions of soybeans. For example, fermented soy foods contain higher levels of free aglycones than other soy-based foods. After isoflavone ingestion, intestinal micro-organisms and enzymes metabolize these compounds in bioactive aglycones such as **1**–**2** and **8** (Table 2) [10].

**Figure 1:**
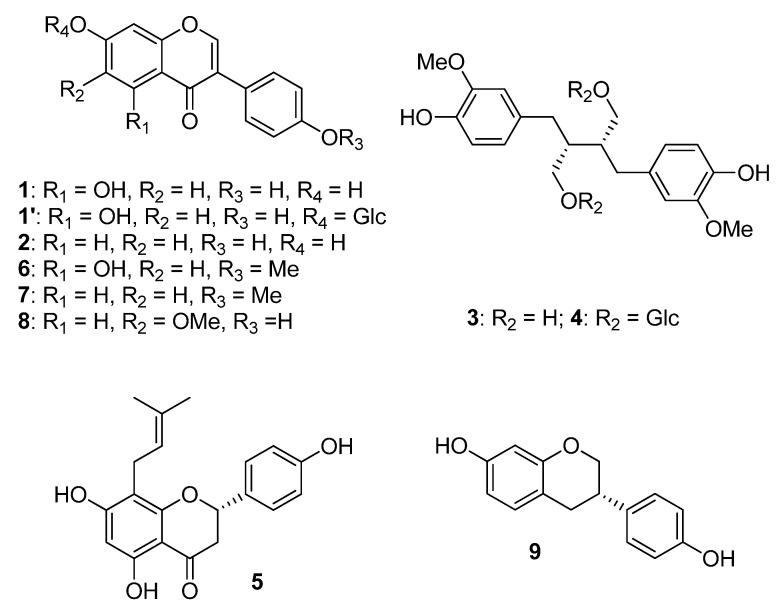
Structure of commonly reported phytoestrogens in food and dietary supplements. Phytoestrogens are polyphenols such as isoflavones (**upper left**) and their metabolites (**bottom right**), enterolignans (**upper right**), or prenylated flavonoids (**bottom left**).

Flaxseed (*Linum usitatissimum* L.) is the richest sources of lignans (60–300 mg of SDG/100 g). In the seed coat, precursors such as the aglycon secoisolariciresinol (SECO, (**3**)) or the glycoside secoisolariciresinol diglucoside (SDG, (**4**)) are present. After ingestion, such lignans are metabolized by the gut microbiota into enterolignans. Like soy isoflavones, the latter are known to exert both estrogenic and non-estrogenic effects. They represent the main dietary intake of phytoestrogens in Western countries. They are also found in sesame seeds (*Sesamum indicum* L.) [12,13]. Hop (*Humulus lupulus* L.) is also a plant known for its hormonal effects. Its main active compound is a flavonoid, i.e., 8-prenylnaringenin (8-PN, (**5**)). Consequently, it is found in beer as well. In contrast with soy isoflavones, 8-PN has a higher affinity for Erα than Erβ [14]. Alfalfa (Medicago sativa L.) and clover (*Trifolium* spp.) are coumestan-rich plants [13] Like soy, red clover (*Triofolium pratense* L.) contains isoflavones. The dominant ones are biochanin A (**6**) and formononetin (**7**), which is metabolized in daidzein. It also contains genistein and daidzein, but in lower quantities compared to soy (Figure 1) [15].

These foods are part of the traditional diet of East Asia. For this reason, isoflavone intake is significantly higher in this part of the world than in Western countries. For example, in Europe, the overall food intake of isoflavones is about 1 mg per day [16], while in Japan, the traditional diet provides 25 to 50 mg of isoflavones per day [17].

In addition to dietary soy, food supplements containing phytoestrogens or plants known to have estrogenic effects are widely marketed to relieve hot flashes usually associated with menopausal disorders as an alternative to hormone replacement therapy. Some examples of dietary supplements containing phytoestrogens available for sale in pharmacies in France are shown in Table 3. These dietary supplements contain various numbers of active compounds, but some do not give precise information on the phytoestrogen dose in their extracts. For those containing soy, information on dosage allows the comparison between studies of the intake from dietary supplements with food intake. None of these food supplements are recommended by the manufacturers for use in women with a personal or family history of BC.

Due to their estrogenic effects, phytoestrogens are handled with caution by the health scientific community from given countries [13] and can cause apprehension, especially for people with a family history of BC. However, in view of the epidemiological data in Asia, it seems reasonable to assume that soy may protect from developing BC. Since the 1990s, many studies have sought to show the link between soy isoflavone consumption and the development of BC. In 2022, the meta-analysis of Boutas et al. concludes that the consumption of soy isoflavones can reduce the risk of BC both in pre- and post-menopausal women [25].

Several in vitro and in vivo studies showed that soy isoflavone aglycones may exert effects that could increase cancer cell development. Some studies on mice models showed that genistein induces MCF-7 cells proliferation in a dose-dependent way. Genistein also induced proliferation of MCF-7 in vitro. The concentration of genistein used in those studies imitated human exposure to dietary isoflavones. Similar results concerning daidzein were found in mice models and in vitro (MCF-7 cells). In one study, genistein specifically triggered the growth in ER+ cells (T47D and MCF-7) but did not impact growth in ER cells. Therefore, daidzein and genistein tumor-promoting effects seem to proceed via ER [26]. Yet, other studies showed beneficial effects of isoflavone aglycones on BC cells and even an anti-cancer treatment perspective. In mice, genistein was able to inhibit cellular matrix metalloproteases (MMP) which are involved in tumor cell migration, potentially alleviating metastasis. Daidzein and genistein can induce apoptosis, including BRCA (breast cancer gene) mutant cancer cells. They also inhibit cytokines and protein kinases. It appears that genistein promotes its anticancer effects via estrogen-independent signaling pathways. Indeed, many genistein properties proceed through its regulation of various genes. Genistein is found to inhibit angiogenesis by reducing circulating levels of VEGF (vascular endothelial growth factor) and decreasing microvascular density [27].

Nevertheless, these in vitro and in vivo data do not allow extrapolating the physiological effects of isoflavones in humans. Indeed, in vitro concentrations are higher than those obtained in vivo, as rodents have plasma levels of genistein aglycone higher than that of humans. These differences between species can be explained by differences in isoflavone metabolism. Indeed, humans have a higher capacity of glucuronidation of steroid-like molecules in the intestine and liver than rats and mice. This allows to maintain unconjugated isoflavones (biologically active form) at relatively low concentrations [28]. Furthermore, estrogen concentration in the breast tissue is much lower than the circulating level [29]. It is therefore more interesting to rely on epidemiological studies.

Such epidemiologic studies were conducted to determine whether there was a protective role of soy isoflavones against BC development. Results from these studies were highly variable. Some found an association between soy isoflavones and a reduction in BC risk, while others did not manage to show this association. However, in general, researchers did not seem to find adverse effects of soy consumption on BC development. In a recent meta-analysis including 16 prospective cohort studies, Zhao et al. found that a high or moderate isoflavone intake, compared to a low one, was not associated with BC risk, whereas a high soy food intake lowers BC risk compared to low soy consumption. Consequently, the mechanism of the preventive role remains unclear [4]. However, this work compared the highest soy isoflavone intake subgroups with the lowest ones from many studies. Not all values were given, and they varied greatly from one study to another. It is therefore difficult to interpret these results. In a meta-analysis of 18 studies, Okenkule et al. assessed that a higher soy intake leads to a reduced risk of BC. More precisely, this association was stronger for ER-negative breast tumors. In post-menopausal women, the study indicated that higher soy consumption reduced this risk, regardless of ethnicity, i.e., in Asian women as well as in other populations [30].

Due to the estrogenic effects of soy isoflavones, one can wonder if the menopausal status could influence the association between soy intake and BC risk. Findings of these studies on the subject were inconsistent and contradictory. In 2014, Chen et al. conducted a meta-analysis to explore the association between soy isoflavone intake and BC risk for pre- and post-menopausal women. They included 30 studies about pre-menopausal women and 31 about post-menopausal women. In pre-menopausal women, soy isoflavone intake seems to be associated with a reduced BC risk. However, when looking more closely, this protective effect was only found in Asian studies versus Western studies. In post-menopausal women, the highest intake of soy isoflavones appeared to reduce BC risk by about 25% compared to the lowest consumption, this association was weaker in Western than in Asian countries [31]. It is important to point out that most of the studies which found an association between soy intake and a decreased risk of BC mostly refer to Asian and not Western populations. This reality could be largely attributed to the difference in the amount of soy consumed in these populations.

A dose–response meta-analysis was conducted on more than 300,000 women to show the impact of soy food intake on BC risk. In this study, the mean value of usual soy isoflavone (aglycone or glycosylated form not specified) intake was 9.4 mg per day, which is equivalent to about 7.5 g of soybeans. Overall, no association between soy intake and BC risk was observed. However, when the studies were stratified by group depending on soy consumption, a decreased risk of BC was found in women, with the highest isoflavone intake (≥40 mg/day) in studies with a global high intake. On the contrary, studies with a global low or moderate soy isoflavone intake found no relation between soy isoflavones and BC risk. Moreover, the meta-analysis did not show any influence of menopausal status on the results. Finally, they assessed that each 10 mg of isoflavones per day was associated with a reduction of 3% of BC risk [32].

In addition to the difference in soy consumption, other factors could explain the stronger benefits on BC risk in Asia than in Western countries. Because soy is part of the traditional diet in East Asia, women are exposed to it from an early age, which seems to have a greater protective effect against BC [33].

In addition, it is unclear whether the same effect in BC risk associated with high soy intake is also applicable to family BC (5–10% of BC cases). In the Korean Hereditary BC Study, Ko et al. studied the impact of soy consumption on BRCA mutation carriers, which represent 25 to 40% of hereditary BC cases. They found that soy product consumption is associated with a lower BC risk in BRCA mutation carriers than in non-carrier family members. This association was stronger for carriers of the BRCA-2 mutation than for the BRCA-1 mutation [34].

A recent study on 76,000 French women over 50 years old examined the relation between the consumption of soy supplements and the risk of BC. Food supplements that were consumed by women contained between 3.75 mg and 37.5 mg of soy isoflavone (glucoside form) per tablet (daily dose not specified). The total follow-up was 11 years. Overall, the study did not find a link between the use of soy supplements and the overall risk of BC. For current users of soy dietary supplements, the results showed a lower risk of ER+ BC. However, they found a higher risk of ER− BC for the current users. This association is stronger for women with a history of BC in first-degree relatives or over 5 years after menopause. The study did not find an association between a past use of soy supplements and BC risk compared with use, whatever the tumors’ ER status. Despite this, some of the authors’ findings led them to advise women with a family history of BC against taking soy supplements based on the precautionary principle [35].

In a 2-year clinical trial, about 400 healthy menopausal women received a daily supplementation of soy isoflavone aglycones in the form of tablets (80 or 120 mg/day), which represented 1 to 4 times a high soy food consumption. This trial study could not conclude that soy isoflavone intake is associated with a decrease in BC risk. However, results were reassuring on the safety of isoflavones for menopausal women. Indeed, the 2-year supplementation did not increase participants’ breast density (which is a risk factor of BC), and no major adverse effects occurred [36].

Based on scientific data at a given moment, various organizations have issued scientific recommendations and opinions regarding soy consumption and the risk of BC development and recurrence.

In France, in 2005, AFSSAPS (Agence française de sécurité sanitaire des produits de santé), i.e., the organization that preceded the current ANSM (Agence Nationale de Sécurité du Médicament), published the report *Sécurité et bénéfices des phyto-estrogènes apportés par l′alimentation—Recommandations*, which contained guidelines about phytoestrogen intake. According to this report, isoflavone-rich foods such as soy foods can be consumed without excess as part of a varied and balanced diet. It is recommended not to exceed 1 mg/kg/day of isoflavones (aglycone equivalents) in food and from dietary supplements. Moreover, particular attention should be applied to pregnant women, post-menopausal women, and those with a personal or family history of cancer. In 2005, according to these experts, the risk of increased tumor cell proliferation could not be excluded. Thus, health authorities recommended specific labeling on dietary supplements containing phytoestrogens (including soy) or fortified foods: “not recommended for women with a personal or family history of BC” [13].

Similarly, in 2015, the European Food Safety Authority (EFSA) published a scientific opinion about the safety of isoflavones from food supplements in menopausal women. Due to limited information, “the Panel did not conclude on the risk of estrogenic isoflavone-based food-supplements in postmenopausal women with a current diagnosis or history of estrogen-dependent cancer”. Furthermore, this assessment did not manage to derive a single health-based guidance value or a safe intake level for food supplements containing isoflavones. The EFSA considers that it is necessary to harmonize the way in which the quantity of isoflavones is presented in food supplement labels [16].

In a different way, in 2012, the American Cancer society concluded in the *Nutrition and Physical Activity Guidelines for Cancer Survivors* that “current evidence suggest no adverse effects on recurrence or survival from consuming soy and soy foods” for BC survivors [37]. The American Institute for Cancer Research even advises to regularly eat soy. They reported that research results differ on whether soy foods are likely to reduce cancer risk, depending on various factors. They added that consistent findings show no increased risk of recurrence or mortality for BC survivors who consume soy foods. They also suggested “greater overall survival and decreased recurrence, among women a year or more after diagnosis who include moderate amounts of soy” [38].

In the United Kingdom, the National Health Service and Cancer Research UK do not give specific recommendations about soy consumption for BC survivors, but they assess that soy may have a weak beneficial effect on BC risk. The NHS mentions that soy intake does not seem to have an impact on BC recurrence [39].

No specific recommendations about soy intake and BC were found for China and Japan. However, soy-based foods are staple foods in the traditional Japanese diet and are commonly consumed in China. The dietary guidelines of these two countries advise Chinese and Japanese residents to eat soy foods daily [40].

The objective of this review consists of analyzing the current literature in order to determine whether it is justified to advise avoiding soy in dietary supplements and/or food in women with a history of BC. Thus, we reviewed studies on the impact of soy intake regarding the mortality and relapse of women with BC.

## 2. Methods

A PRISMA (Preferred Reporting Items for Systematic reviews and Meta-Analyses) approach was applied to conduct a systematic literature search [41,42]. Both Medline/Pubmed and Web of Science databases were used as information sources. Using Pubmed, specific MeSH terms were selected and searched to identify papers published between 2009 and 2020 as follows: “breast neoplasm” AND (“soybeans” OR “soy foods” OR “isoflavones”). Using Web of Science, the following keywords were searched to select articles issued in the same period: “soy” AND “breast cancer”; “isoflavones” AND “breast cancer”. Regarding the selection process, articles included in this review meet the following criteria: only full articles were retained, and editorials and reviews were excluded. We selected only prospective cohort studies. The endpoint of studies was BC recurrence and/or mortality and the association with soy isoflavone intake specifically identified.

## 3. Results

We identified a total of 11,566 results (7826 on PubMed and 3740 on Web of Science). After exclusion of duplicates and results that did not meet selection criteria, seven studies were selected for this review (Table 4).

In their study, Guha et al. subdivided the inputs of isoflavone aglycones into the following components: genistein (**1**), daidzein (**2**) and glycetin (**8**). The intakes of **1** and **2** seemed to be highly collinear. They found a non-significant decreased risk of BC recurrence with an increased intake of **1**–**2** and **8**. This association was stronger for post-menopausal women. A linear trend was almost statistically significant. When the analysis of results was stratified by hormonal receptor status, the study showed that results vary for the association between glycetin (**8**) intake and BC recurrence, but not for genistein (**1**) and daidzein (**2**) intakes. For women who were treated with tamoxifen, the risk of recurrence decreased when the glycetin (**8**) intake increased, and this association was statistically significant. The same association was not significant for both genistein (**1**) and daidzein (**2**). For never-users of tamoxifen, there was no beneficial association between cancer recurrence and a high isoflavone intake. In the 95th percentile of isoflavone intake there was even an almost significant increased risk of recurrence. The association of daidzein (**2**) intake and BC recurrence was evaluated in women who had never used tamoxifen and were menopausal. This risk was significantly lower in the highest category of daidzein (**2**) intake compared to the lowest [43].

Woo et al. studied the link between legumes and soy isoflavone intake and BC recurrence related to human epidermal growth factor receptor 2 (HER2) status. They concluded that high isoflavone intake (highest vs. lowest terciles) were inversely associated with BC recurrence in women with HER2- BC, whereas a high intake of isoflavones (highest vs. lowest terciles) slightly increased or did not change (statistically non-significant) the risk recurrence for HER2+ BC [44].

In the Shanghai BC Survival Study, women in the highest soy food intake groups had the lowest mortality and recurrence rate compared with women in the lowest soy food intake group. This association seems to follow a linear dose–response relation up to 40 mg/day of soy isoflavones. Beyond that, this association seems to reach a plateau. Further analysis of the results shows that the association is observed in women with ER-positive or ER-negative BC. It did not appear to have differences between post-menopausal and pre-menopausal women. A high soy isoflavone consumption (highest quartile) combined with tamoxifen use did not appear to improve this treatment efficacy over the reduction in BC recurrence [45].

Kang et al. specifically studied the effects of soy isoflavones on BC recurrence and death for patients receiving adjuvant endocrine therapy and were affected by ER+ and/or PR+ cancer. The results showed no effect among pre-menopausal women, and the effect did not vary when analyzing hormonal receptor status. In post-menopausal women, an inverse association was found between soy isoflavones and BC recurrence. Indeed, women in the highest quartile of soy isoflavone intake (>42.3 mg/day) had a significantly lower risk of recurrence than those in the lowest quartile. There was no effect of soy isoflavone intake on mortality in post-menopausal women. The associations found on mortality were similar for BC-specific mortality and all-cause mortality. Post-menopausal women with ER+/PR+ BC that were in the highest quartile of isoflavone intake had a significantly lower risk of recurrence than those in the lowest intake quartile. Post-menopausal patients receiving anastrozole therapy in the highest quartile of isoflavone intake (>28.83 mg/day) had a lower risk of recurrence than those in the lowest [46].

In the Conroy et al. study, pre-diagnosis soy intake was not related to a decreased all-cause or BC-specific mortality. They did not find any evidence of a dose–response association. When stratified by type of cancer, there was no evidence that hormonal receptor status influences the association between soy intake and breast-cancer-specific mortality. They conducted an analysis by ethnicity and found weak differences between the subgroups. A slight decrease in mortality was observed only for Native Hawaiian participants. The comparison between other subgroups did not yield significant results [47].

Zhang et al. conducted a study on dietary isoflavone intake and all-cause mortality in BC survivors. They concluded that a higher isoflavone intake (>1.5 mg/day) was associated with a decrease in all-cause mortality (21%) when compared to women with a lower consumption. This result was statistically significant for post-diagnosis intake but weaker and not statistically significant for intake before the diagnosis of BC. It was also significant in women with ER−PR− tumors and without hormonal therapy but not in women with hormone-receptor-positive tumors. When comparing the main dietary isoflavones, there were no differences in results between genistein (**1**), daidzein (**2**), and glycetin (**8**) [48].

**Table 4:** Characteristics of studies on the association between soy food intake and BC.

Study (Author, Year of Publication and Country)	Cohort Size; Events; Duration of Follow-Up (in Years)	Soy Intake	Results (HR, CI95%) of Soy Isoflavone Aglycone Intake: mg/Day; Soy Product Intake/Soy Protein Intake: g/Day
LACE study (Guha et al., 2009, USA) [43]	n = 1954; 282 recurrences; average follow-up = 6.31 y	Semi-quantitative FFQ and soy FFQ Mean daidzein intake = 1.7 mg/day Mean genistein intake = 2.4 mg/day	highest (>9.54) vs. lowest (<0.15) terciles of soy intake based on daidzein distribution daidzein intake/recurrence: 0.96 (0.52–1.76) genistein intake/recurrence: 0.95 (0.52–1.75) glycetein intake/recurrence: 0.80 (0.42–1.50)
SBCSS (Shu et al., 2009, China) [45]	n = 5033; 444 deaths and 534 recurrences; average follow-up = 3.9 y	FFQ No average intake value indicated	highest vs. lowest quartiles soy protein intake (H > 15.31 vs. L < 5.31) total mortality: 0.67 (0.51–0.88) recurrence: 0.66 (0.52–0.84) soy isoflavone intake (H > 62.68 vs. L < 20) total mortality: 0.76 (0.58–0.99) recurrence: 0.74 (0.59–0.95)
(Kang et al., 2010, China) [46]	n = 524; 154 deaths (132 BC); average follow-up = 5.1 y	FFQ Mean soy isoflavone intake = 25.6 mg/day (aglycone or glycoside, form not specified)	highest (>42.3) vs. lowest (<15.2) quartiles SII total mortality: 1.05 (0.78–1.71) pre and 0.88 (0.56–1.24) post recurrence: 0.88 (0.61–1.23) pre and 0.67 (0.54–0.85) post
(Woo et al., Korea, 2012) [44]	n = 339; 25 recurrences; average follow-up = 32.6 months	FFQ No average SII intake value indicated Mean intake of soy black beans = 6.22 g/day	highest vs. lowest terciles recurrence-free/survival soy products (H > 65.7 vs. L < 36.2): 0.95 (0.41–2.20) isoflavones (H > 15.2 vs. L < 7.4): 0.75 (0.30–1.90)
(Zhang et al., 2012, China) [49]	n = 616 recurrences and 79 cancer-related deaths; average follow-up = 52.1 months	FFQ Mean soy isoflavone aglycone intake = 17.32 mg/day	highest (>28.83) vs. lowest (<7.56) quartiles of SII total mortality: 0.62 (0.42–0.90)
(Conroy et al., USA, 2013) [47]	n = 3842; 804 deaths (376 BC-related); average follow-up = 6.2 y	Quantitative FFQ Median daily intake: 3.7 mg isoflavones/1000 kcal	highest (>10.4) vs. lowest (<4.3) terciles of soy isoflavones BC-specific mortality: 1.01 (0.74–1.39) total mortality: 0.98 (0.79–1.39)
(Zhang et al., USA, 2017) [48]	n = 6235; recurrences and 1224 deaths; average follow-up = 9.4 y	FFQ Mean soy isoflavone intake = 1.8 mg/day	highest (>1.494) vs. lowest (<0.342) quartiles of SII pre-diagnosis intake: 0.84 (0.66–1.06) post-diagnosis intake: 0.65 (0.41–1.00)

LACE: Life After Cancer Epidemiology; SBCSS: Shanghai BC Survival Study; FFQ: food frequency questionnaire; SII: soy isoflavone intake.

In their study, Zhang et al. found that a high intake of soy isoflavone was inversely associated with BC mortality. A soy isoflavone aglycone intake (sum of **1**–**2** and **8**) above 17 mg per day seems to reduce BC mortality by about 36% compared to a low consumption. However, no linear dose–response association was found between soy food intake and BC survival. This tendency is also shown by the study of high soy protein intake. When the analysis is stratified by ER status, the study found a better prognosis in women with ER-positive BC and a high soy isoflavone intake, but the difference with ER-negative BC was weak [49].

## 4. Discussion

While many studies were conducted on the link between soy and BC risk, only seven recent publications meeting all criteria, i.e., prospective cohort studies about the impact of soy phytoestrogens on BC recurrence and/or mortality, were found after methodical research. Selected studies for this work present some limitations. The cohort size was very variable but most of the time rather limited, especially when compared with studies that evaluated the association between soy intake and BC development. There is a probable reporting bias of soy isoflavone intake which is inherent in dietary studies. All selected studies used a food frequency questionnaire and most of the time it was self-administered by participants of the study. This could have led to imprecisions in values but allowed classification in subgroups of soy isoflavone intake, i.e., low, medium, and high intake. This imprecision is particularly present when the pre-diagnosis intake is evaluated. Finally, the total durations of the follow-ups are relatively short and variable between studies. For this work, the mean follow-up is 5.42 years. This could prevent the evaluation of long-term effects of soy on BC outcomes by limiting the number of events.

Overall, only one study was not able to find the beneficial effects of soy intake on BC patients [47]. The other studies concluded that there were positive associations but in very variable ways. Indeed, Guha et al. and Kang et al. found a decrease in BC recurrence associated with a higher isoflavone intake only in post-menopausal women [43,46]. The other four studies [44,45,48,49] concluded that there were positive associations regardless of menopausal status. Several studies [43,44,46,49] showed better results in women with hormonal sensitive cancer and/or patients receiving hormonal treatment. Only Zhang et al. [48] found a stronger association for patients with ER-negative BC. No selected study showed a positive association only for pre-menopausal women.

None of these studies found statistically significant adverse effects of soy consumption on BC recurrence or mortality (specific or all-cause).

In their review and meta-analysis, Qiu et al. concluded that pre-diagnosis soy isoflavone intake was associated with a statistically significant decrease in all-cause mortality and recurrence of BC, and these effects were mainly observed for post-menopausal women. They were not able to find an association between soy intake and BC-specific survival [17].

Another meta-analysis from Nechuta et al. [50] brought together three cohorts (LACE, WHEL, and SBCSS) from the United States and China, which totals about 9500 BC survivors. They concluded that soy food consumption after diagnosis greater or equal to 10 mg of soy isoflavones per day was associated with a statistically significant reduction in recurrence and a non-significant reduced risk of all-cause and BC-specific mortality. This association was found among Chinese and American patients even when Asian-American women were excluded. In this article, US women had a mean consumption of 3.2 mg of soy isoflavones per day and Chinese women had a mean consumption of 45.9 mg of soy isoflavones per day.

The wide difference in soy isoflavone intake between Asian studies (China, Japan, and Korea) and Western studies is found in most studies. It could explain the differences in results between studies. Indeed, in general, beneficial effects of the association between soy isoflavone intake and BC recurrence and/or mortality are more often found in Asian studies than in Western ones. This observation could suggest that there is a dose–response relationship for soy isoflavones. Some studies investigated this hypothesis. Zhang et al. did not manage to find a linear dose–response regarding the association, but they showed an increased trend in the survival rate with an increased intake of soy isoflavones [49]. Shu et al. found a positive association which appeared to follow a linear dose–response effect up to a soy isoflavone intake of 40 mg per day, and then it leveled off [45]. Therefore, the dose of isoflavones consumed seems to have major importance in the positive effects that soy may have on BC outcomes.

The mechanisms that could explain the beneficial effects of soy isoflavones on BC recurrence and mortality are unclear and complex. As mentioned above, phytoestrogens can bind to estrogen receptors due to their structural similarity with 17β-estradiol. Two main types of ERs have been identified in humans: ERα and ERβ. A link to subtype α of these receptors has been shown to increase the cell proliferation necessary for growth and maintenance of the tissues, but it can also play a role in the unlimited growth of ER-dependent breast tumors. The subtype β has an opposite effect: it neutralizes the ERα-mediated stimulation of cell proliferation. The distribution of these subtypes varies according to the types of tissues, which could influence the cellular response to different phytoestrogens. Indeed, experimental studies have shown that phytoestrogens (and particularly daidzein or genistein, on which most studies are based) have less powerful estrogenic effects than endogenous 17β-estradiol. Furthermore, they have a preferential selectivity for ERβ compared to Erα, whereas 17β-estradiol, at a physiological concentration, may only activate ERα. For genistein, studies indicate that concentrations that activate ERα will at the same time activate ERβ. That will counteract the ERα-mediated effects on cell proliferation. Thus, phytoestrogens act like SERM; overall, they exert anti-estrogenic effects that could inhibit the proliferation of BC cells and explain the beneficial role on BC outcomes [6].

Other properties of phytoestrogens may provide additional support for a reduced risk of developing and relapsing BC. Indeed, phytoestrogens are likely to exert non-estrogenic effects. Since inflammation has a key role in cancer recurrence and prognosis, the anti-inflammatory and antioxidant properties known for soy may provide additional benefits among the inverse association between soy intake and BC recurrence and mortality [51]. Other modes of action have been described in vitro, and it has been shown that soy isoflavones increase the synthesis of sex-hormone-binding globulin, inhibit kinase, and increase the clearance of steroids from circulation. Thus, this lowers the availability of these hormones [6,52]. Genistein seems to have epigenetic effects that could explain the potential effects on lowering BC recurrence or mortality. That may influence the regulation of cell proliferation and differentiation. For example, in vitro, genistein has effects on DNA methylation, as it lowers the methylation of several tumor-suppressor genes and allows them to be re-expressed. This mechanism might explain why the incidence of BC is lower in certain Asian populations. However, these effects on methylation can also affect tumor-suppressor genes and then have a potential adverse effect [6].

After menopause estrogen production stops in ovaries, the only sexual hormones that are formed come from the peripheral tissues and exert their effects locally. The concentration of 17β-estradiol in the tumor is several times higher than in blood circulation or normal breast tissue. That suggests a specific tumoral synthesis of estrogens from circulating precursors. Experimental studies have shown the presence of all the enzymes responsible for the local biosynthesis of estrogens in BC tissues by two main pathways: the aromatase pathway and the sulfatase pathway. It is interesting to notice that soy isoflavones inhibit the activity of aromatase and 17β-hydroxysteroid dehydrogenases responsible for the synthesis of estradiol from estrones and androgens. Therefore, it seems appropriate to assume that isoflavones may inhibit the local production of estrogen from circulating precursors in breast tissue. That could explain the greater benefit of soy isoflavone intake on BC recurrence and/or mortality among post-menopausal women compared to pre-menopausal women in several studies [53].

However, it is not always possible to make a link between mechanisms involving phytoestrogens highlighted by in vitro or animal studies and the results found in human observational studies. For example, HER2 (human epidermal growth factor receptor 2) stimulates tyrosine kinase activity, which leads to cell proliferation. In vitro, in some studies, genistein (1) has an inhibition of tyrosine kinase activity which inhibits HER2 gene overexpression and is involved in apoptotic cell death and cell cycle arrest. In theory, isoflavone soy intake including 1 might have beneficial effects on HER2-positive BC. Other studies considering the interaction between 1 and BC treatment found opposite results. In their study, Woo et al. found a positive association between soy intake and risk of BC recurrence only in women with HER2-negative BC. For women with HER2-positive BC, they did not find a significative association or even a non-statistically significant slight increased risk [44].

Moreover, we can wonder if there are other factors in addition to soy isoflavones that could impact BC patients’ risk of recurrence and mortality. Indeed, as previously mentioned, some factors are known to impact these risks: alcohol or tobacco, characteristics of tumor, menopausal status, BMI (body mass index), physical exercise, etc.

In several studies, a higher soy consumption seemed to be associated with a “healthy lifestyle”, i.e., a diet rich in vegetables, fish and plant-based protein and a low intake of fat and red meat, in addition to more physical exercise [45,48,49]. Studies showed that high vegetable intake and regular physical activity may be related to better survival, but evidence remains rather inconsistent and there is a need for further investigation [54,55].

Most selected studies adjusted their results to possible confounding factors, including, for some of them, healthy habit factors such as diet and physical activity [45,47,48]. For example, Shu et al. take into account cruciferous vegetable intake, red meat intake, fish intake, any vitamin supplement use, tea consumption, and physical activity [45]. This could allow to limit errors in estimation of the association between soy food intake and BC recurrence and mortality.

As mentioned above, the intestinal microbiome plays a role in the metabolism of phytoestrogens in the gastrointestinal tract. In plants, isoflavones are present mainly in a glycosylated form and need to be transformed into aglycone compounds to exert their effects. Therefore, it is essential that the glycosidic bond is cleaved in the small intestine. Daidzein (2) is metabolized by the microbiome in *S*-equol (9) (which has a higher estrogenic effect) or O-desmethylangolensin. The bacterial flora is known to be highly variable between individuals, so the production of active estrogenic compounds from ingested phytoestrogens might be too. The production of 9 can be considered as an indicator of the potential effects of soy on human health, and some individuals might not be able to produce active compounds [56]. These potential individual differences might lead to the very variable effects of soy isoflavones between women. This could influence the global results of studies. Overall, it seems that *S*-equol has more anticancer than oncogenic effects, although future studies should specify the required concentration of 9 [57].

Although soy is the main source of phytoestrogens in several Asian countries, its consumption remains generally low in Western countries. In these countries, the dietary intake of phytoestrogens can come from other plants. Moreover, some women consume dietary supplements that must be considered in the total daily intake of phytoestrogens. They are taken to relieve hot flashes associated with menopause symptoms as an alternative to hormone replacement therapy. For example, in Europe, the phytoestrogen food intake is mainly composed of lignans (about 58–67%), and isoflavones represent only 30–38% [58]. These other phytoestrogen sources may also exert an effect on the risk of BC recurrence or mortality.

Flax is a food commonly used in dietary supplements. It is the richest source of lignans and particularly of SDG (4). Data found in literature suggested the efficacy of flax to relieve hot flashes, but the results remain quite inconsistent. Some works showed that flax lignans could exert antitumor effects, but few studies were conducted on the effects of flax on BC. Observational studies suggested that lignan consumption may decrease the risk of BC. However, data available on the subject are quite limited, especially on flax safety for women with BC [12].

Like soy, red clover is a plant containing isoflavones. It is used in dietary supplements for relieving menopausal disorder. In the literature, few studies evaluated the efficacy of red clover extract on menopausal disorders. Results seemed positive but not homogeneous, and no major side effects were detected during these trials. However, the studies had very few samples, and patients were cancer-free [15,59].

Hop is also used in dietary supplements because of the estrogenic effects of 8-PN (5). Few studies were conducted and were mainly about the effects of hop or hop extract on hot flashes. Their results suggested that hop might reduce these symptoms (vs. placebo). However, there is a need for larger investigation to assess hop effects and safety, especially on BC survivors. There is a special concern about 5 because, unlike soy isoflavones, it has a higher preference for ERα. Therefore, it could have different effects than isoflavones [14,60].

Thus, few studies have been published on these plants rich in phytoestrogens. Therefore, limited data are available on the safety of products containing these herbal medicines, especially for BC survivors. Unlike isoflavones, there is no quantitative recommendation for the intake of other phytoestrogens in the general population or for women with BC history.

No study specifically evaluating the use of soy isoflavone supplements in BC survivors has been found. However, it seems reasonable to refer to the results of studies in which the mean soy isoflavone intake is high, such as in Asian studies, because amounts consumed by women in the highest groups of soy intake are approaching those present in some dietary supplements (Table 3). For example, Shu et al.’s study had the highest quartile of soy isoflavone intake, greater than 62.68 milligrams per day [45]. Kang et al. had a mean soy isoflavone aglycone intake of 25.6 milligrams per day and the highest quartile, greater than 42.3 milligrams per day [46]. According to these studies, the results seemed to be reassuring about the safety of isoflavones for BC survivors.

## 5. Conclusions

In conclusion, in this review, none of the selected studies found statistically significant adverse effects of soy consumption on BC recurrence or mortality (specific or all-cause). Almost all studies showed beneficial effects of soy isoflavone intake, in variable ways, on BC recurrence and mortality. These results coincide with other recent works and suggest that soy isoflavone intake is safe for BC survivors.

However, in order to confirm the current results, larger studies are needed. Future research should have larger sample sizes, more accurate assessments of soy isoflavone intake (and total phytoestrogen intake), better control of confounding factors, and longer follow-ups.

## Figures and Tables

**Table 1:** Binding parameters of some phytoestrogens to ER.

	IC_50_(nM)		Hill Coefficient	
Compounds	ERα(9)	ERβ(10)	ERα(9)	ERβ(10)
17β-estradiol	2.8	4.06	1.67	1.07
genistein	17	12.5	0.94	1.03
daidzein	230	23.7	0.80	0.62
biochanin A	1800	352	0.92	0.77

**Table 2:** Isoflavone contents of soy foods [11].

Type of Food	Total Isoflavones in Aglycone Equivalents (mg/100 g)
Soybeans	104
Tofu	27.1
Tempeh	18.3
Textured vegetable protein (soy based)	16.1
Miso paste	11.2
Soy yoghurt	10.2
Soy milk	2.9
Soybeans sprouts	0.8

**Table 3:** Examples of phytoestrogen dietary supplements. * Aglycone or glycoside form not specified.

Name	Phytoestrogen Sources	Daily Dose–Composition Dosage (per Day)	Extract Daily Dose (mg/Day)	Phytoestrogen Daily Dose (mg/Day)
Phyto Soya^®^ 17.5 mg—Arkopharma [18]	Soy	2–4 capsules	soy: 350–700	35–70 isoflavones = 22–44 aglycone equivalents
Phyto Soya^®^ ménopause—Arkopharma [19]	Soy	2 capsules	soy: 250	70 isoflavones = 44 aglycone equivalents
Ergyflavone^®^—Nutergia [20]	Soy and hop	2 capsules	soy: 250 hop *: 60	50 isoflavones *
Sojyam^®^—Nutreov [21]	Soy	1 capsule	soy: 150	60 isoflavones *
Menophytea^®^ équilibre—Nutreov [22]	Flax, hop, and kudzu	2 day capsules 2 night capsules	flaxseed *: 210 hop *: 100 kudzu *: 50	
Triolinum^®^ jour/nuit—Nutreov [23]	Flax and hop	2 day capsules 2 night capsules	hop *: 300 flaxseed: 80 hop *: 100 flaxseed: 20	16 SDG 4.6 SDG
Triolinum^®^ fort +—Nutreov [24]	Flax and hop	2 capsules	hop *: 40 flaxseed: 250	50 SDG
